# Deep learning-based image segmentation for predicting hot carcass weight in tropical beef cattle

**DOI:** 10.1007/s11250-026-04920-2

**Published:** 2026-02-24

**Authors:** Gutierrez José de Freitas Assis, Nathalia Farias de Souza, Erica Beatriz Schultz, Cris Luana de Castro Nunes, André Henrique Franco Costa, Antônio Almeida Santos Neto, José Augusto Miranda Nacif, Lucas Bragança da Silva, Ricardo dos Santos Ferreira, Mario Luiz Chizzotti

**Affiliations:** 1 Department of Animal Science, Federal University of Viçosa, Av. Peter Henry Rolfs, s/n - Campus Universitário, Viçosa, Minas Gerais Brazil; 2 Department of Science and Technology Institute, Federal University of Viçosa - Campus Florestal, Rodovia LMG 818, km 06, s/n, Campus Universitário, Florestal, 35.690-000 Minas Gerais Brazil; 3 Department of Informatics, Federal University of Viçosa, Av. Peter Henry Rolfs, s/n - Campus Universitário, Viçosa, Minas Gerais Brazil

**Keywords:** Bovine, Computer vision, Machine learning, Precision livestock

## Abstract

The application of computer vision and deep learning in the meat processing industry enables automated carcass evaluation. This study aimed to develop and validate a deep learning-based pipeline for automatic carcass segmentation and prediction of hot carcass weight (HCW) in tropical beef cattle. A total of 598 RGB images of bovine half-carcasses were collected under commercial slaughterhouse conditions and manually annotated to delineate carcass boundaries. For segmentation, a YOLOv11 model was trained. From the segmented images, geometric and shape descriptors were extracted and subsequently used in a LASSO regression model to predict HCW. A strong segmentation performance was achieved, with an Intersection over Union (IoU) of 0.92 and a Precision of 0.98. For HCW prediction, the model achieved R² = 0.84 and MAPE = 5.77%. The integration of deep learning–based segmentation with regularized regression provides a practical and scalable approach for carcass evaluation. The combination of computer vision and statistical learning enables real-time, accurate prediction of beef carcass weight.

## Introduction

Accurate determination of carcass weight is essential in beef processing, influencing pricing, yield calculations, and supply chain management (OECD/FAO, 2021). Although industrial scales provide straightforward measurements of carcass weight, they offer limited information on carcass composition, conformation, and meat quality traits. More detailed assessments require specialized phenotyping methods, such as imaging technologies or genetic evaluations (Khanal et al. 2019; Leighton et al. 2022). Computer vision and machine learning offer scalable alternatives capable of estimating weight alongside morphological shape descriptors, supporting automated inspection, auditing, and traceability in commercial slaughterhouses (Allen 2021; Barbar et al. 2022; Delmore 2022).

The use of imaging technologies in the meat industry has drawn attention for over two decades, with early studies such as Sonnichsen et al. (1998) exploring image-based classification and prediction of quantitative and qualitative traits in beef carcasses. More recently, Wakholi et al. (2022) demonstrated that deep learning techniques can effectively extract visual features and integrate them with machine learning algorithms to achieve up to 90% precision in predicting hot carcass weight (HCW). Nisbet et al. (2025) demonstrated that image-based approaches are not only capable of accurately predicting cold carcass weight in cattle but can also be applied at different processing stages to assess carcass conformation and fat cover. Regarding the broader potential of this technology, Matthews et al. (2022) further highlighted its ability to go beyond carcass weight prediction by enabling the estimation of cut yield and supporting product traceability along the meat production chain.

These advances are particularly relevant, however, their application to carcass evaluation has been scarcely explored, especially in Brazilian slaughterhouses, despite the leading role in beef production across tropical regions.

Among deep learning architectures, You Only Look Once (YOLO) stands out for its remarkable balance between speed and accuracy, enabling rapid and reliable object detection and segmentation in images, and making it particularly well suited for real-time applications (Terven et al. 2023). To date, YOLO has not been applied to segmentation of tropical beef carcasses, representing a novel opportunity for precision meat science.

This study aims to (1) develop a YOLO-based model for delineating beef carcass regions from RGB images and (2) evaluate morphometric shape descriptors derived from segmentation for predicting the HCW of beef cattle under tropical abattoir conditions.

## Materials and methods

### General information

All pre-slaughter and slaughter procedures complied with Brazilian regulations (MAPA, 2021). The study was conducted in a commercial beef processing facility, where 598 half-carcass images were acquired from beef cattle raised under tropical conditions with diverse genetic backgrounds, sexes, ages, and HCW, ensuring a representative sample of commercial variability. An RGB camera (Intel^®^ RealSense™) was installed at the end of the slaughter line to capture side-view one frame of each half-carcass at 1280 × 720-pixel resolution, providing sufficient detail for subsequent segmentation analysis.

### Dataset preparation

**Fig. 1:**
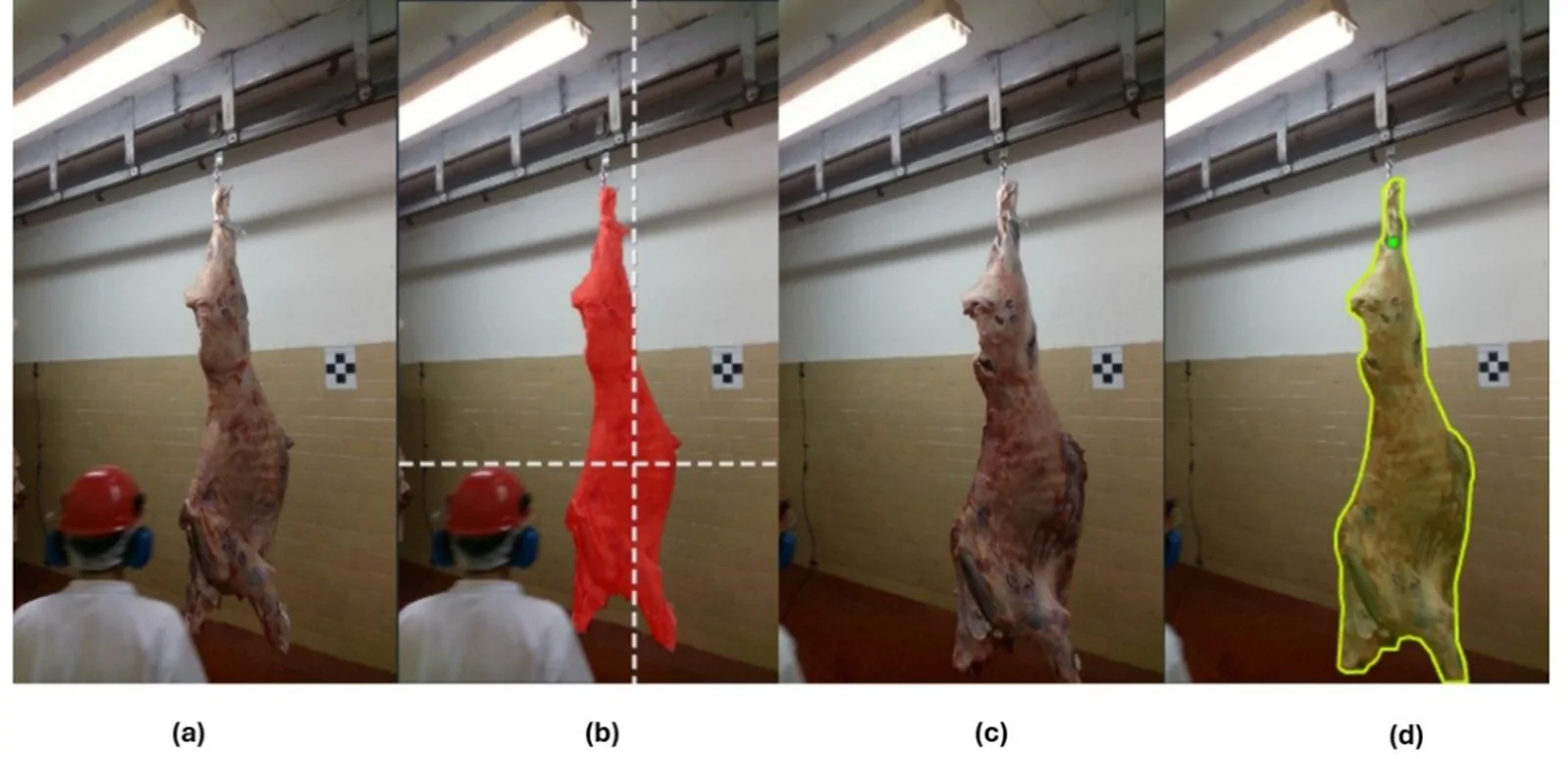
Examples of the annotation process performed in Roboflow, illustrating (**a**) and (**c**) the original image, (**b**) the smart polygon tool used for region of interest (ROI) delimitation, and (**d**) the original image with the corresponding segmentation mask applied to beef carcass images

A custom dataset was developed to train the YOLO version 11 segmentation model, with precise annotations of carcass regions. Annotation and preprocessing were performed using the Roboflow platform (Fig. 1), enabling creation of both bounding boxes and segmentation masks while streamlining the labeling process. The dataset was randomly split into 418 training images (70%) and 180 validation images (30%) to ensure robust model evaluation. In the YOLO segmentation workflow, each object includes two representations: bounding boxes for after localization and segmentation masks for precise delineation of carcass contours.

### Model architecture

The segmentation task was performed using the YOLO architecture (Ultralytics library, version 11.0.0) with transfer learning from the pretrained ‘yolo11-seg.pt’ checkpoint, enabling faster convergence and improved accuracy. Training was conducted with over 100 epochs with a batch size of 16, as this number of epochs was sufficient to ensure convergence of the loss function and stabilization of performance metrics, while avoiding overfitting. Model performance was evaluated using standard segmentation metrics: Intersection over Union (IoU), Precision and Recall (Powers 2020).

### Feature extraction

Morphological and geometric descriptors were extracted from segmented carcass masks to quantify their structural and spatial characteristics (Table 1) as reported by Gonzalez and Woods (2008).

**Table 1:** Summary of shape descriptors extracted from carcass segmentation masks

Shape Descriptor	Description
Area (px²)	The total number of pixels within the segmented region, representing the surface area of the carcass in the image.
Perimeter (px)	The length of the boundary of the segmented region, measured in pixels.
Convex Hull Area (HConvex) (px²)	The area of the smallest convex polygon that encloses the segmented region, used to assess compactness.
Solidity (-)	The ratio of the Area to the Convex Hull Area; values close to 1 indicate a more convex shape, while lower values suggest concavity or irregularity.
Aspect Ratio (AR)	The ratio of the bounding box width to its height, providing a measure of elongation or overall shape orientation.
Extent (-)	The ratio of the Area to the area of the bounding box; it indicates how much of the bounding box is filled by the segmented region.
Equivalent Diameter (ED) (px)	The diameter of a circle that has the same area as the segmented region. This is useful for approximating carcass size with a single scalar value.
Circularity (-)	A shape metric defined as$\:4\pi\:\:x\:Area/Perimeter$^2^; it ranges from 0 to 1, where 1 indicates a perfect circle.
Eccentricity (-)	A measure of how much the shape deviates from a circle, calculated as the ratio between the distance of the foci of the ellipse and its major axis length.
Orientation Radians (OR) (-)	The angle (in radians) between the major axis of the ellipse that fits the shape and the horizontal axis, indicating the orientation of the carcass.
Major Axis Length (Major) (px)	The length of the longest line that can be drawn through the region (based on an ellipse fit), often associated with carcass length.
Minor Axis Length (Minor) (px)	The length of the line perpendicular to the major axis (ellipse fit), typically representing carcass width.
Elongation (-)	Calculated as the ratio of Major to Minor axis lengths, indicating how stretched or narrow the shape is.
Centroid X (CX) (px) and Centroid Y (CY) (px)	The (x, y) coordinates of the center of mass of the region, often used for spatial alignment or positional reference.

(-): admensional; px: pixel

### Statistical analysis

#### Pearson correlation

Extracted shape descriptors were analyzed via Pearson correlation to explore their relationships with HCW. These analyses were conducted in RStudio version 2023.12.1 (R Core Team, 2023).

#### Regression models

The dataset, combining selected morphometric shape descriptors and corresponding HCW, was used to train three regression models: Least Absolute Shrinkage and Selection Operator (Lasso), Random Forest (RF), and Adaptive Boosting (AdaBoost). Lasso regression employed L1 regularization to perform simultaneous shrinkage and variable selection. All predictors were standardized prior to model fitting, and the regularization parameter (λ) was optimized using 10-fold cross-validation by selecting the value that minimized the cross-validated mean squared error. RF regression was implemented as an ensemble of decision trees to capture non-linear relationships and enhance robustness to multicollinearity, with key hyperparameters tuned through cross-validation. AdaBoost regression combined weak learners in a sequential manner to minimize prediction errors, with model hyperparameters also selected using cross-validation. All regression models were trained using an 80:20 training/validation split, and performance was assessed using coefficient of determination (R²), mean absolute error (MAE), root mean square error (RMSE), and mean absolute percentage error (MAPE). Model development and evaluation were carried out in Python (version 3.11) using the scikit-learn library.

## Results

### Segmentation model performance

**Fig. 2:**
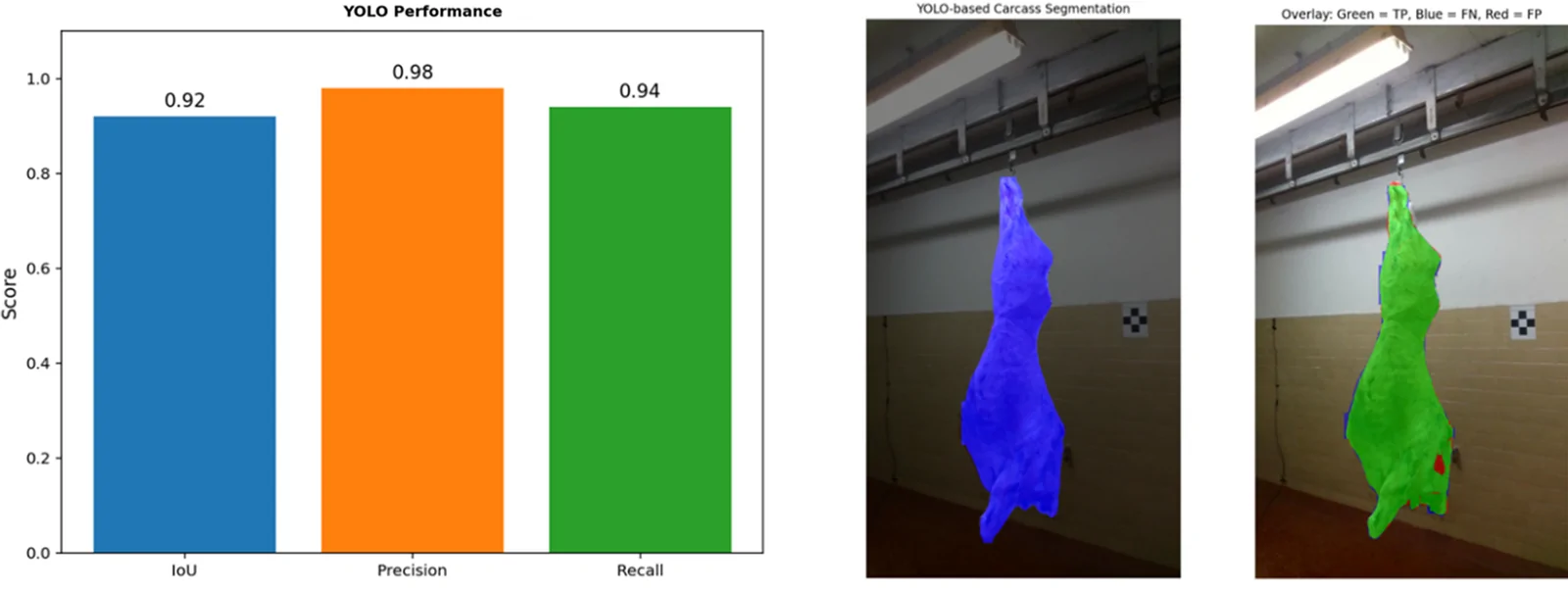
Segment yolo-based model and Carcass Inference. IoU: Intersection Over Union. Left: Original image with model inference, illustrating segmentation of the carcass including internal regions. Right: Color overlay highlighting true positives (green), false negatives (blue), and false positives (red)

The YOLO model achieved high precision (> 0.90) in the segmentation of beef carcasses from tropical cattle (Fig. 2). Detection errors were mainly observed along the carcass edges.

### Feature extraction from segmentation masks

#### Descriptive statistics of shape descriptors

Table 2 shows that carcass weight ranged from 66.9 to 216.1 kg, providing the dataset with substantial variability, which is crucial for ensuring model generalizability, particularly in weight prediction tasks.

**Table 2:** Descriptive statistical analysis of final dataset

Feature	Mean	SD	Min	Max
Hot Carcass Weight, kg	154.66	27.19	66.90	216.10
Area, px²	122,710.6	18,449.63	55,312.00	183,584.00
Perimeter, px	2,278.49	128.12	1,648.43	2,564.78
Convex Hull Area, px²	166,993.85	23,422.94	76,848.00	241,210.00
Solidity, (-)	0.73	0.02	0.67	0.80
Aspect Ratio, (-)	0.29	0.03	0.21	0.36
Extent, (-)	0.53	0.03	0.45	0.61
Equivalent Diameter, px	394.11	30.33	265.38	483.47
Circularity, (-)	0.30	0.02	0.22	0.36
Eccentricity, (-)	0.97	0.01	0.95	0.98
Orientation Radian, radius	0.01	0.04	-0.08	0.15
Major, px	880.18	59.54	663.39	1,033.36
Minor, px	216.20	20.83	126.67	273.27
Elongation, (-)	4.09	0.33	3.33	5.73
Centroid X, px	365.63	69.06	151.05	539.33
Centroid Y, px	872.58	23.04	764.38	923.57

(-): admensional; px: pixel

#### Pearson correlation analysis

**Fig. 3:**
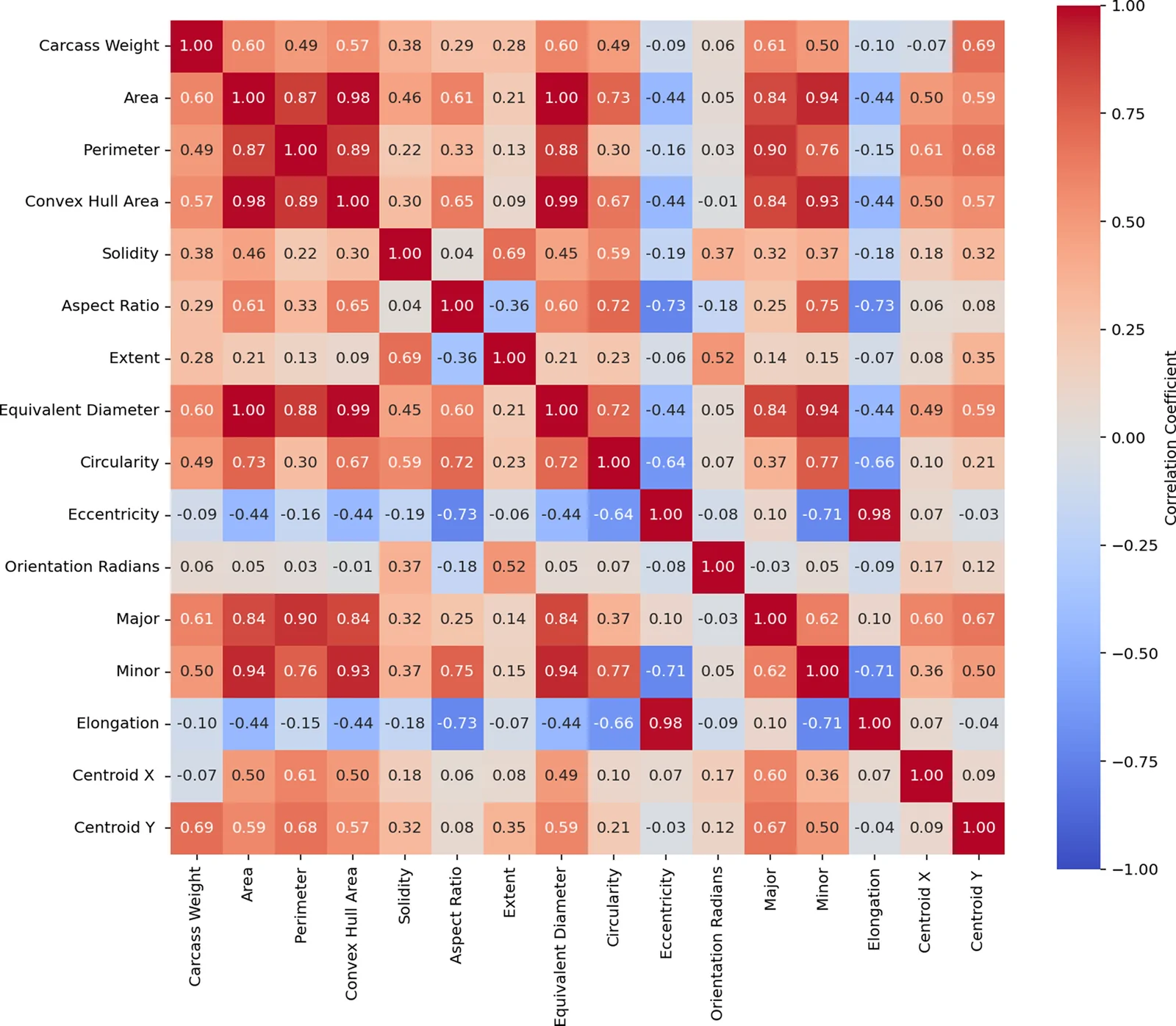
Correlation matrix of the morphological shape descriptors derived from the segmentation dataset. The heatmap illustrates the Pearson correlation coefficients between feature pairs, highlighting strong positive and negative relationships. High correlation values indicate potential multicollinearity and redundancy among shape descriptors, underscoring the need for careful feature selection in subsequent modeling steps

Pearson correlation analysis revealed strong associations among size-related morphological shape descriptors. Mainly for area, major and Centroid Y ≥ 0.60. The lowest correlated descriptor was eccentricity ≤ 0.10. There was a high correlation between the most morphological shape descriptors of image (Fig. 3).

### Regression model performance for weight prediction

**Fig. 4:**
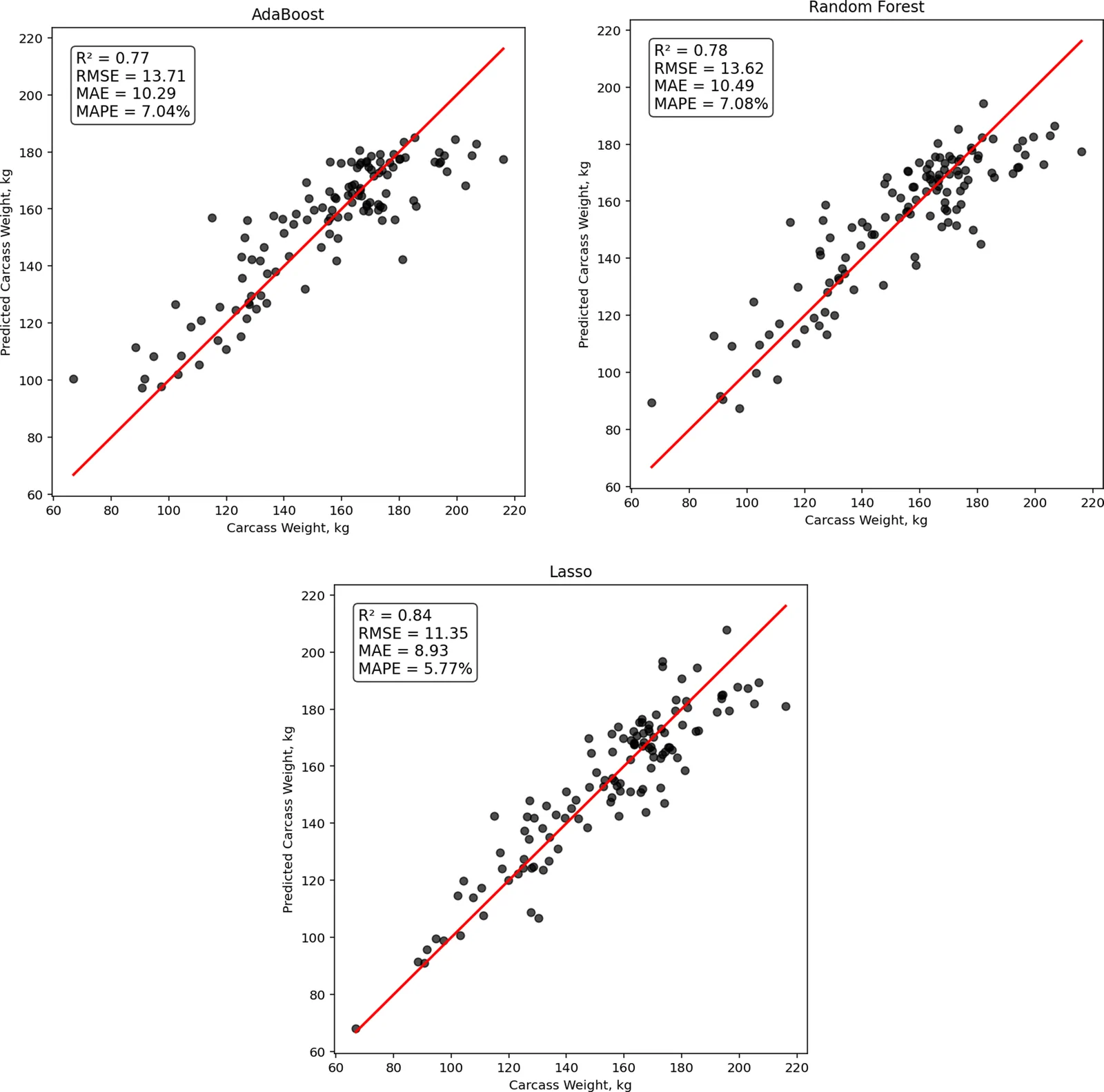
Regression models performance in predicting the hot carcass weight

All models demonstrated strong predictive performance, with R² values exceeding 0.75. The Random Forest and AdaBoost models achieved R² values of 0.78 and 0.77, respectively, while the Lasso model reached 0.84. Corresponding error metrics also indicated reliable predictions: the RMSE ranged from 11.35 to 13.71 kg, and the MAE ranged from 8.93 to 10.49 kg, showing consistent accuracy in estimating carcass weight (Fig. 4).

The top 90% variables are mainly associated with the position, size, and geometric shape of the carcass. They reflect the morphometric aspects most directly related to weight, whereas the remaining 10% have a lower influence (Table 3).

**Table 3:** Presents the variables that were more important in each model

Model	90% Importance Variable	10% Importance Variable
RF	Centroid Y, Circularity, Centroid X, Major, Orientation Radians, Elongation, Convex Hull Area, Equivalent Diameter	Area, Perimeter, Extent, Minor, Aspect Ratio, Solidity, Eccentricity
AdaBoost	Centroid Y, Major, Centroid X, Circularity, Elongation, Convex Hull Area, Orientation Radians	Equivalent Diameter, Area, Perimeter, Extent, Aspect Ratio, Minor, Solidity, Eccentricity

RF: Random Forest

## Discussion

### Model performance and accuracy

The YOLO segmentation model demonstrated robust performance for carcass evaluation, achieving high IoU, precision, and recall (Figs. 2). These results confirm its reliability in accurately delineating carcass boundaries and anatomical structures across diverse beef samples, even in challenging slaughterhouse environments. Previous studies have applied CNN-based segmentation to 2D images for carcass evaluation, such as Gonçalves et al. (2020), who used VGG-19 for preliminary segmentation of Zebu carcasses, and Wakholi et al. (2022) for Hanwoo cattle, highlighting the potential of deep learning for predicting carcass yield. Our work is the first to combine 2D images with YOLO specifically for tropical beef cattle carcasses. The combination of spatial precision and fast inference positions YOLO as a practical tool for real-time deployment in commercial slaughterhouses, supporting automated inspection and grading by minimizing false positives. With appropriate adjustments, smartphone cameras could also be used for HCW estimation, enhancing accessibility and scalability. While alternative architectures can offer higher segmentation detail, they often incur higher computational costs; future studies could explore hybrid, or ensemble approaches to balance accuracy and efficiency.

### Correlation analysis and shape descriptors relationships

Correlation analysis (Fig. 3) revealed moderate to strong associations between size-related descriptors—such as Area, Equivalent Diameter, Major Axis, and Convex Hull Area—and carcass weight, consistent with the expectation that heavier carcasses occupy larger projected areas in 2D images. Centroid Y also showed notable correlation, whereas shape descriptors like Eccentricity, Elongation, and Orientation exhibited weak correlations. High correlations among shape descriptors such as Area, Perimeter, Convex Hull Area, Equivalent Diameter, Solidity, and Major/Minor axes indicated multicollinearity, this redundancy can inflate coefficient variances, reduce model stability, and hinder interpretability in linear regression models (Arora and Dhir 2017).

### Evaluation of regression model performance

The three regression models demonstrated satisfactory predictive performance for estimating HCW based on 2D morphometric shape descriptors. Among them, Lasso regression outperformed the ensemble methods, achieving the highest predictive accuracy. Lasso regression performs variable selection through L_1_ regularization, which effectively reduces the influence of less informative or highly correlated shape descriptors. By retaining only the most relevant morphometric descriptors, Lasso produced a simpler and more interpretable model, minimizing overfitting and improving generalization. During training, a 10-fold cross-validation was applied to optimize the model and ensure robust variable selection, with all folds used solely on the training data. The final evaluation was then performed on the independent test set, providing an unbiased assessment of predictive performance (Obuchi and Kabashima 2016). This approach has likely contributed to the superior results observed by Lasso compared to Random Forest and AdaBoost, where all predictors are retained and model interpretability can be lower.

These shape descriptors primarily represent carcass position, size, and geometric shape, highlighting their central role in weight prediction. Negative coefficients observed for shape descriptors such as Perimeter may reflect biological realities, where more compact or irregular carcass shapes correspond to variations in muscle distribution or fat deposition, resulting in slightly lower weights. In contrast, Lasso retained positive coefficients for key dimensions and orientation descriptors, reinforcing their direct contribution to predicting carcass weight.

The predictive performance achieved using 2D morphometric shape descriptors combined with Lasso in this study demonstrates that a relatively simple imaging approach can provide robust estimates of carcass weight. For comparison, Nisbet et al. (2024) applied machine learning algorithms, including Random Forest and Artificial Neural Networks, to predict cold carcass weight in cattle using 3D images, extracting 44 morphometric shape descriptors. While our study used 2D shape descriptors, the results show that this approach is feasible and practical, particularly for tropical breeds and slaughterhouse settings where 3D imaging may be less accessible.

Thus, it could be concluded that the integration of YOLO-based segmentation with regularized regression demonstrates the technical feasibility of non-invasive carcass weight estimation of beef cattle raised under tropical conditions. This framework could be implemented in slaughterhouse computer vision systems to support automated inspection, facilitating data-driven decision-making, process standardization, and the adoption of precision technologies in meat processing, ultimately improving efficiency, objectivity, and scalability.

## Data Availability

The datasets analysed during the current study are available from the corresponding author upon request.

## References

[CR1] Allen P (2021) Recent developments in the objective measurement of carcass and meat quality for industrial application. Meat Sci 181:108601. 10.1016/j.meatsci.2021.108601 34182344

[CR2] Arora A, Dhir R (2017) Pearson correlation-based feature selection for chromosome shape analysis. Med Biol Eng Comput 55(6):897–906. 10.1007/s11517-016-1553-2 27474041

[CR3] Barbar C, Bass PD, Barbar R, Bader J, Wondercheck B (2022) Artificial intelligence-driven automation is how we achieve the next level of efficiency in meat processing. Anim Front 12(2):56–63. 10.1093/af/vfac017 35505849 PMC9056041

[CR5] Delmore RJ (2022) Automation in the global meat industry. Anim Front 12(2):3–4. 10.1093/af/vfac021

[CR6] Gonçalves DN, de Moraes Weber VA, Pistori JGB, da Costa Gomes R, de Araujo AV, Pereira MF, Gonçalves WN, Pistori H (2020) Carcass image segmentation using CNN-based methods. Inform Process Agric. 10.1016/j.inpa.2020.11.004

[CR7] Gonzalez RC, Woods RE (2008) Digital Image Processing (3rd ed.). Prentice Hall, Upper Saddle River, NJ, USA. ISBN: 978-0131687288

[CR9] Khanal P, Maltecca C, Schwab C, Gray K, Tiezzi F (2019) Genetic parameters of meat quality, carcass composition, and growth traits in commercial swine. J Anim Sci 97(9):3669–3683. 10.1093/jas/skz247 31350997 PMC6735811

[CR10] Leighton PL, Segura J, Lam S, Marcoux M, Wei X, Lopez-Campos O, Soladoye P, Dugan ME, Juarez M, Prieto N (2022) Prediction of carcass composition and meat and fat quality using sensing technologies: A review. Meat Muscle Biology 5(3):12951. 10.22175/mmb.12951

[CR11] Matthews D, Pabiou T, Evans RD, Beder C, Daly A (2022) Predicting carcass cut yields in cattle from digital images using artificial intelligence. Meat Sci 184:108671. 10.1016/j.meatsci.2021.108671 34656003

[CR555] Ministério da Agricultura, Pecuária e Abastecimento (2021) Portaria SDA/MAPA nº 365, de 16 de julho de 2021: Regulamento Técnico de Manejo Pré-abate e Abate Humanitário e métodos de insensibilizaçáo autorizados. Diário Oficial da Uniáo Brasil

[CR12] Nisbet H, Lambe N, Miller GA, Doeschl-Wilson A, Barclay D, Wheaton A, Duthie C-A (2024) Machine learning algorithms for the prediction of EUROP classification grade and carcass weight using 3-dimensional measurements of beef carcasses. Front Anim Sci 5:1383371. 10.3389/fanim.2024.1383371 38043328

[CR13] Nisbet H, Lambe N, Miller GA, Doeschl-Wilson A, Barclay D, Wheaton A, Duthie CA (2025) On-farm 3D images of beef cattle for the prediction of carcass classification traits and cold carcass weight. Animal 101529. 10.1016/j.animal.2025.101529 40409162

[CR14] Obuchi T, Kabashima Y (2016) Cross validation in LASSO and its acceleration. J Stat Mech: Theory Exp 2016(5):053304. 10.1088/1742-5468/2016/05/053304

[CR15] OECD/FAO (2021) OECD-FAO Agricultural Outlook 2021–2030. OECD Publishing, Paris, France. 10.1787/19428846-en

[CR16] Powers DM (2020) Evaluation: From precision, recall and F-measure to ROC, informedness, markedness and correlation. arXiv preprint arXiv:2010.16061

[CR222] R Core Team (2023) R: A language and environment for statistical computing (Version 4.3.0). R Foundation for Statistical Computing. https://www.R-project.org/

[CR18] Sonnichsen M, Augustini C, Dobrowolski A, Brandscheid W (1998) Objective classification of beef carcasses and prediction of carcass composition by video image analysis. In: Proceedings of the 44th International Congress of Meat Science and Technology, pp. 30–34

[CR19] Terven J, Córdova-Esparza DM, Romero-González JA (2023) A comprehensive review of YOLO architectures in computer vision: from YOLOv1 to YOLOv8 and YOLO-NAS. Mach Learn Knowl Extr 5(4):1680–1716. 10.3390/make5040083

[CR20] Wakholi C, Kim J, Nabwire S, Kwon KD, Mo C, Cho S, Cho BK (2022) Deep learning feature extraction for image-based carcass yield Estimation. Biosyst Eng 218:78–93. 10.1016/j.biosystemseng.2022.04.008

